# Comparison of citrate dialysate in pre- and post-dilution online hemodiafiltration: effect on clot formation and adequacy of dialysis in hemodialysis patients

**DOI:** 10.1080/0886022X.2024.2302109

**Published:** 2024-01-08

**Authors:** Pasu Nakornchai, Arisara Jitraree, Monpinya Charttong Homjan, Thanachit Laykhram, Thananda Trakarnvanich

**Affiliations:** Division of Nephrology, Department of Medicine, Faculty of Medicine, Vajira Hospital, Navamindradhiraj University, Bangkok, Thailand

**Keywords:** Adequacy of dialysis, citrate dialysate, clot formation, hemodialysis, pre-dilution, post- dilution, online hemodiafiltration

## Abstract

**Background:**

Citrate dialysate (CD) has been successfully used in conventional hemodialysis and continuous renal replacement therapy; however, no study has compared pre- and post-dilution online hemodiafiltration (oL-HDF). Therefore, we aimed to investigate the efficacy of citrate anticoagulation for oL-HDF and the metabolic changes and quality of life of patients on hemodialysis treated using both modes

**Method:**

Eight dialysis patients were treated with CD containing 0.8 mmol of citric acid for 4 weeks in each phase. Visual clotting scores were investigated as the primary endpoints. Adequacy of dialysis, laboratory parameters, and quality of life were measured as secondary objectives.

**Results:**

The mean clotting scores in the pre-dilution mode were significantly lower than those in the post-dilution mode and in all phases except the heparin-free phase (*p* < 0.001 in the baseline phase, *p* = 0.001 in phase 1, and *p* = 0.023 in phase 2). The values of Kt/V in both modalities were comparable except during the baseline phase, in which the values of pre-dilution were significantly greater than post-dilution (2.36 ± 0.52/week vs. 1.87 ± 0.33/week;95% CI −0.81 to −0.19, *p* = 0.002). The patient’s quality of life regarding their physical activity level was significantly higher in the post-dilution mode than in the pre-dilution mode at baseline and in phase 1 (*p* = 0.014 and 0.004 at baseline and in phase 1, respectively). Metabolic changes did not differ between the two modes.

**Conclusion:**

Citrate dialysate decreased or prevented anticoagulation in both pre- and post-dilution modes of oL-HDF without significant side effects and had comparable adequacy of dialysis.

## Introduction

Online hemodiafiltration (oL-HDF) is being increasingly used in patients undergoing chronic dialysis because of its improved convective clearance. The removal of large or protein-bound uremic retention solutes can be achieved, in addition to the excellent clearance of small molecules [1]. Furthermore, oL-HDF has been associated with better hemodynamic tolerance and biocompatibility and may even result in better survival [2] with reduction of proinflammatory cytokines [3,4] and circulating cells [5], as well as better control of beta-2 microglobulin (β_2-_MG) levels [6]. The oL-HDF is classified into two types according to the mode of addition of the substitution fluid: pre-dilution oL-HDF (pre-HDF) and post-dilution oL-HDF (post-HDF). Post-HDF is the most effective way to maximize molecule clearance, with higher removal rates of β_2_-MG and α_1_-microglobulin (α1-MG), albeit significantly higher albumin leakage, than hemodialysis (HD) and pre-HDF[7]. However, a limitation of post-HDF is the intermittent hemoconcentration between the dialyzer and venous drip chamber, which can cause thrombosis. On the other hand, pre-HDF can resolve this problem [8], but requires approximately three times more purified water than post-HDF and may not elicit maximal clearance.

Heparin is the most routinely used anticoagulant in hemodialysis (HD). Although the complication rates with heparin are low, certain complications such as bleeding and thrombocytopenia can occur. Furthermore, long-term use of heparin induces biological and clinical complications (osteoporosis, dyslipidemia, and hyperkalemia) [9–11]. This has led to the exploration of alternative anticoagulants in patients with end-stage kidney disease (ESKD). Regional citrate anticoagulation is a good alternative to heparin use in HD for patients with increased bleeding risk [12], but its implementation is cumbersome [13,14]. Citrate dialysate (CD) has proven to be a safe alternative to acetate-containing dialysate in dialysis without significant hypocalcemia or clinically important coagulation issues [15–17]. Therefore, the present study aimed to compare the patency of the dialyzer, quality of life (QOL), and blood parameters between pre- and post-HDF CD patients. To date, no study has compared the use of CD between these two modalities in oL-HDF.

## Materials and method

This trial was prospectively registered at ClinicalTrials.Gov Identifier: NCT05280106 (14/03/2022) and was approved by the Institutional Review Board of the Faculty of Medicine, Vajira Hospital,Navamindradhiraj University,Bangkok,Thailand (COA 080/64). The study was conducted in accordance with the Declaration of Helsinki and Good Clinical Practice guidelines, and all study methods were carried out in accordance with relevant guidelines and regulations. All patients who participated in the study signed an informed consent form before enrollment.

In this prospective one-group interrupted time series design (ITS), eight adult chronic dialysis patients (five women and three men) were already treated with HD at our dialysis unit three times per week. These included three men and five women; their mean age was 63.38 ± 17.00 years (range 38–86 years). These patients had been in a chronic HD program for a median of 8.05 years (range 3.12–14.7 years). The inclusion criteria were as follows: age 18–80 years, stable condition (normal blood pressure, no signs of infections, and no signs of other serious illnesses), and HD for at least 3 months. Vascular access should have a blood flow ≥ 300 mL/min. Exclusion criteria were no heparin use during HD, refusal to participate, intolerance or allergy to citrate, concurrent infection, poor life expectancy due to metastatic cancer, severe cirrhosis, hemorrhagic diseases, and acquired immunodeficiency syndrome (AIDS). Patients with vascular access modification, those affected by chronic liver disease, those with active neoplastic or inflammatory diseases, and those receiving immunosuppressives, anti-inflammatory drugs and calcimimetics were excluded.

The study was performed using a Fresenius 5008 dialysis machine (Fresenius Medical Care, Bad Homburg, Germany) or Nikkiso DBB-05 machine. All patients underwent HD three times per week for 4 h in each session using a high-flux polyester polymer alloy dialyzer with an effective surface area of 2.1 m^2^ (FDY21 GW, Nikkiso, Japan). The blood flow rate was maintained at 300–350 mL/min, individualized to the patient’s vascular access, to achieve an effective convection rate. The dialysate flow (Qd) rate was maintained at 500 mL/min. The dialysate sodium concentration was 140 mmol/L and bicarbonate concentration was 35 mmol/L. The heparin dose intradialysis ranged from 500 units in 4 h to 5,000 units at the maximum dosage and was carried out according to standard procedures continously throughout the dialysis session.Citrate acid concentrate contained 0.8 mmol of citric acid and 0 mmol of acetic acid; standard concentrate consisted of 3 mmol of acetic acid and 0 mmol of citrate. The dialysis prescription was aimed at obtaining effective convective exchanges of > 20 L/session. The CD contained 0.8 mmol/L citric acid and 1.25–3.5 mmol/L calcium (adjust according to the calcium levels of the patients). The dialysis fluid purity utilized in this study met the International Organization for Standardization criteria for ultrapure water and dialysate, indicated by total viable microbial counts of less than 0.1 colony forming units (CFU)/mL, and endotoxin concentrations of less than 0.03 EU/mL. The water and dialysate samples were tested monthly for biological contamination. Patients were not allowed to consume food or take antihypertensive medications during dialysis. Erythropoiesis stimulating agents (ESA) and iron were adjusted to keep the level of hemoglobin (Hb) in the range 10–13 g/dL, transferrin saturation more than 20%. Phosphate binders such as calcium carbonate and non-calcium based regimens were prescribed to keep serum calcium and phosphorus within normal limits. Vitamin D supplement was given to keep total vitamin D level more than 20 ng/mL.

The spKt/V was used to quantify dialysis adequacy using the second-generation single-pool Daugirdas formula (Kt/V = -ln (R-0.03) * [(4–3.5)* R) * (UF/W)], where R = post-dialysis blood urea nitrogen (BUN)/pre-dialysis BUN, UF = net ultrafiltration, W = weight, K = dialyzer clearance of urea, t = dialysis time, and V = patient’s total body water.

We also assessed QOL in both modalities using the short form of the Kidney Disease Quality of Life (KDQOL), that is, the KDQOL-short form (SF) [18]. It is a self-report measure that includes an SF item health survey as the generic core and multi-item scales targeted on kidney disease and dialysis, including the burden of kidney disease, symptoms and problems with kidney disease, and effects of kidney disease. The KDQOL-SF can be split into generic and disease-specific parts. The domains of the SF-36 can be summarized in two summary scores: physical functioning (physical component summary [PCS]) and mental functioning (mental component summary [MCS]). The mean score of the general United States population was 50, with a standard deviation of 10 [19]. The disease-specific part of the KDQOL-SF consisted of 44 kidney disease-targeted questions. The short KDQOL (KDQOL-SF™), currently version 1.3 [18], consists of 36-item of SF-36 and 11 domains of the kidney disease-targeted domain including symptoms/problems (12 items), effects of kidney disease on daily life (8 items), burden of kidney disease (4 items), work status (2 items), cognitive function (3 items), quality of social interaction (3 items), sexual function (2 items), and sleep (4 items). It also included multi-item measures of social support (two items), dialysis staff encouragement (two items), and patient satisfaction (one item). These domains are scored from 0 to 100, with higher scores indicating an absence of problems [19].

## Study protocol

**Figure 1.**
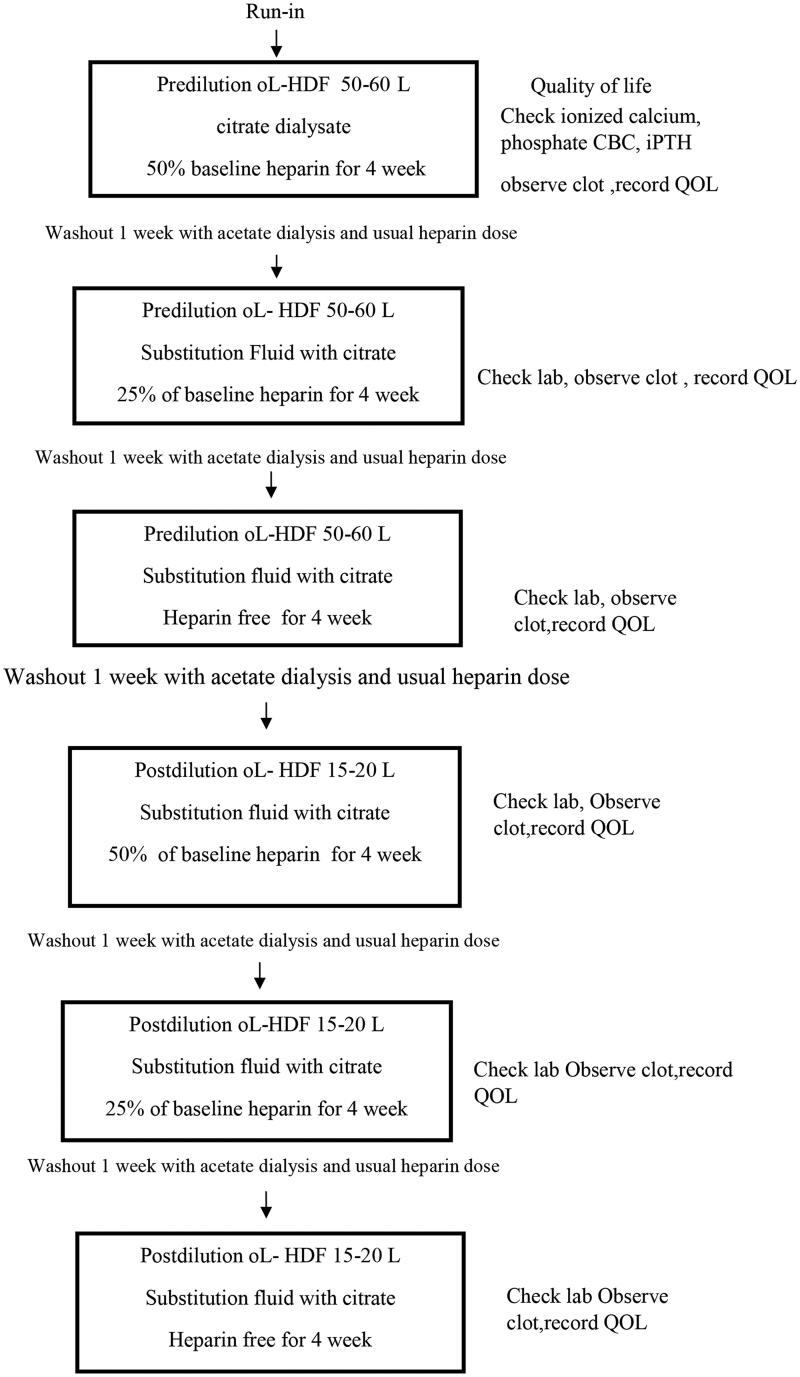
Study flow chart.

The study was conducted over a 29-week period in two phases. For the first 12 weeks, all patients were subjected to CD with pre-HDF. After enrollment, all patients began a 2-week run-in period with conventional intermittent hemodialysis (IHD) fluid containing acetate and the lowest dose of unfractionated heparin. We titrated the heparin dose by reducing the dose step-by-step and 25% in each step until the final minimal dose was achieved. The experimental study consisted of three phases, with four weeks in each phase, and the phases were separated by 1-week washout periods using online HDF. Phase 1 used CD with a 50% heparin dose at baseline. In phases 2 and 3, 25% heparin and no heparin were used, respectively. New dialyzers were introduced at the beginning of each phase and the heparin dose was resumed during the washout phase. The study design is illustrated in Figure 1. The calcium concentration in the dialysis fluid was maintained at constant (1.5 mmol/L) to evaluate the effect of CD on calcium balance, followed by the same protocol with post-HDF in the same patients for the following 12 weeks.

In both modalities, we assessed QOL and the end of each phase and recorded the case record forms.

### Data collection

Baseline clinical and laboratory parameters were determined at the beginning of the run-in period—the beginning of each treatment period (pre- and post-HDF period). Blood samples were collected to analyze the complete blood count, electrolytes, intact parathyroid hormone (iPTH), calcium, phosphorus, and albumin levels at the beginning of each phase. Ionized calcium (iCa) was measured pre- and post-HD in each session. The single-pool Kt/V was measured using the second-generation equation of the Daugirdas formula. The dialyzer clotting was assessed using the corrected visual scale adapted from Aniort et al. [17] (Table 1).

**Table 1.** Restitutional clotting scale.

Clotting score	External appearance of venous chamber
0	No or very little residual blood in the filters
1	Residual blood in less than 10% of the fibers
2	Residual blood 10–25%
3	Residual blood 25–50%
4	Residual blood > 50%

Adapted from: [17].

### Quantification of dialysis dose

To compare the dialysis doses of the two online modalities, the effective convection volume normalized to a body-size-related factor was used as a surrogate marker [20]. The effective convection volume is the total volume of the undiluted fluid that is ultrafiltered during treatment, including the fluid removed for weight loss. In post-HDF, the effective convection volume is equal to the total ultrafiltered volume, including weight loss. The ultrafiltration volume must be adjusted for the degree of dilution using a dilution factor (DF) that adjusts the effect of upstream infusion on the concentration of solute in the ultrafiltrate. The DF was calculated based on the total plasma water volume processed divided by the total non-erythrocyte water volume passed through the dialyzer (plasma water plus upstream infused fluid) as follows:

$$\text{Qpw}=\text{Qb}*\left(1-\text{Hct}\right)*1\left(0:016\;\text{Pct}\right)$$

$$\text{DF}=\frac{\text{Qpw}}{\text{Qpw}-\text{Qinf}}$$

$$\begin{array}{l} \text{Qpw}\;=\;\text{plasma water flow rate},\;\;\\ \text{Qinf}\;=\;\text{flow infused upstream},\;\\ \text{Qb}\;=\;\text{blood flow rate},\;\\ \text{Hct}=\;\text{hematocrit},\text{ and Pct }=\text{ rit} \end{array}$$

The protocrit is the volume fraction of plasma proteins, which can be calculated as the product of 0.000718 and the total protein concentration of plasma proteins in g/mL.

## Calculating the UF rate to be used in pre-dilution

We planned to balance the effective convection flow rate (Qfeff) between pre- and post-dilution modes. We then calculated the ultrafiltration rate in pre-dilution (Qfpre) by multiplying by a factor of 1/DF such that Qfpre = Qfeff/DF. In pre-dilution HDF, the actual filtration rate needs to be at least 30–50% of the blood flow entering the dialyzer to achieve an effective convection rate of at least 20% of the undiluted blood flow rates.

## Dialyzer reprocessing procedure

The dialyzer reprocessing procedure involved four steps: rinsing, cleaning, performance testing, disinfection, and sterilization. All steps were manually reused following the guidelines developed by the Association for the Advancement of Medical Instrumentation (AAMI):

1. The reprocessing area should be a separate area with good ventilation.
2. Return the blood using the machine’s blood pump and 0.9% normal saline. Air should not be allowed to enter the blood tubings or the dialyzer. Fill the circuit adding 1000 U of heparin to the saline before disconnecting it completely from the patient.
3. Remove the dialyzer and tubings from the machine and take to the reprocessing area in a covered tray to avoid blood spills. The tubings should be disconnected and the blood compartment of the dialyzer connected to the water source. The blood compartment should be rinsed with water till the effluent is clear.
4. Dialyzers should be labeled carefully and always used for the same patient. The external of the dialyzers should also be inspected that the caps are well tightly secured at both ends.
5. Clean by instilling 1% hypochlorite into the blood compartment till it is completely filled and allow the blood compartment to remain filled for up to two minutes. Immediately rinse out of the cleaning agent from the blood compartment.
6. The one end of the blood compartment is connected to the water supply, which is turned off, whereas the other end is left open. The one end of the dialysate compartment is capped, whereas the other is connected to a water supply with a pressure of 1–1.3 bar through a Hansen’s connector. The water should enter the dialysate compartment and exit through the blood compartment. The direction of flow should be reversed at five-minute intervals.
7. The dialyzer should be tested to make sure there are no broken fibers and it is still working.
8. The dialyzer is filled with peracetic acid
9. When the dialyzer is ready for use, paracetic acid is rinsed out.
10. The dialyzer is tested to make sure no germicide is left and the dialyzer can be used safely by measuring total cell volume (TCV), which should always be at least eighty percent compare to baseline according to the National Kidney Foundation’s Kidney Disease Outcomes Quality Initiative.

## Clotting scores

The clotting scores were stratified according to the scales shown in Table 1.

### Sample size calculation

For the study design with one dependent sample, a crossover study with the same group of patients with CD underwent pre- and post-dialysis [21]. The main outcome was the clotting score for each of the five levels. If we used the clot score at 2 h with mean score = 4.3 (standard deviation [SD] = 0.7) and after HDF mean score = 4.0 (SD = 1.2), the effect size = 0.5, α = 0.05, β = 0.2 (power 0.8). The number of HDF was 35 sessions, calculated using program G Power 3.1.9.4.

### Statistical analysis

Data are expressed as mean ± SD for normally distributed data and median (IQR) for non-normally distributed data. The difference between the two modalities at each time point was assessed using a paired t-test (normally distributed data) and Wilcoxon signed-rank test (not normally distributed data). Parametric tests were performed using the one-way Kolmogorov–Smirnov test. Repeated analysis of variance (ANOVA) was used if there were more than two independent variables. All *p*-values were one-tailed, and *P*-values less than 0.05 were considered to indicate statistical significance. The Friedman test was used to determine the effects of CD on the clotting score, QOL scores, and Kt/V in each phase. Differences were considered statistically significant at *p* < 0.05 (two-tailed test). All statistical tests were performed using the Statistical Package for the Social Sciences (IBM SPSS version 2).

## Results

### Patient characteristics

In total, 13 patients were recruited. Three were excluded owing to calcimimetic consumption, and the remaining 10 patients were enrolled. Eight patients completed the study, and two terminated the study prematurely due to death from sepsis. The baseline patient characteristics are shown in Table 2. The mean age of the patients was 68.38 ± 17.00 years. The etiologies of chronic kidney disease (CKD) included diabetes mellitus (*n* = 3), presumed chronic glomerulonephritis (*n* = 4), and IgA nephropathy (*n* = 1). The mean body mass index (BMI) was 23.43 ± 5.54 kg/m^2^. Nearly all patients (*n* = 6,75.00%) used native arteriovenous fistula (AVF), while two cases used perm catheters.

**Table 2.** Baseline characteristics.

Characteristics (*n* = 8)		
	n	%
age (yrs.), Mean ± SD (Min - Max)	68.38 ± 17.00 (38–86)	
sex		
F	5	62.50
M	3	37.50
BMI (kg/m^2^), Mean ± SD (Min - Max)	23.43 ± 5.54 (18.29–31.56)	
DM	3	37.5
HT	8	100.00
CAD	3	37.50
vascular access		
AVF	6	75.00
permanent catheter	2	25.00
cause of ESRD		
CGN	4	50.00
DKD	3	37.50
IgA	1	12.50
gout	2	25.00
dialysis vintage (yr), Median (IQR)	8.05 (3.12–14.7)	

SD: standard deviation; BMI: body mass index; CGN: chronic glomerulonephritis; DKD: diabetic kidney disease; DM: diabetes mellitus; HT: hypertension; CAD: cardiovascular disease; AVF.

Treatment characteristics between both modalities were comparable, except for substitution volume and Qfeff (Table 3). In pre-HDF, the mean substitution volume was 42.81 ± 5.06 L, while the volume was 18.27 ± 2.28 L in the post-dilution mode. Consequently, adding an ultrafilter volume to the substitution volume resulted in an effective convection volume. To achieve the target effective convection volume, the ultrafiltration rate in the pre-dilution mode must increase by a factor of 1/DF. The mean effective convection volume of pre-dilution was greater than in the post-dilution mode (23.06 ± 1.20 L vs. 20.60 ± 1.68 L, *p* = 0.002).

**Table 3.** Treatment characteristics.

Qb convection								
Outcome	Pre phase	Phase 1 heparin 50%		Phase 2 heparin 25%		Phase 3 heparin-free		
	Mean ± SD/Absolute difference	*p* Value	Mean ± SD/Absolute difference	*p* Value	Mean ± SD/Absolute difference	*p* Value	Mean ± SD/Absolute difference	*p* Value
**blood flow**								
Predilution	291.93 ± 9.86		295.31 ± 6.71		297.25 ± 3.91		298.37 ± 3.65	0.079^c^
Postdilution	296.09 ± 5.75		301.44 ± 9.91		294.43 ± 20.45		301.47 ± 11.64	0.307^c^
Change from pre to post (95% CI)^a^	4.17 (-3.08 to 11.41)	0.260	6.13 (-1.12 to 13.37)	0.097	−2.82 (-10.07 to 4.42)	0.445	3.1 (-4.14 to 10.34)	0.402
Difference from heparin-free (95% CI)^b^	1.07 (-8.51 to 10.65)	0.827	3.03 (-6.55 to 12.61)	0.535	−5.92 (-15.5 to 3.65)	0.225	Ref.	
**Substitute volume**								
Predilution	41.97 ± 4.20		40.93 ± 4.06		43.71 ± 6.09		43.79 ± 6.27	0.181^c^
Postdilution	45.61 ± 5.57		18.37 ± 2.41		17.59 ± 2.87		18.85 ± 2.16	<0.001^c^
Change from pre to post (95% CI)^a^	3.64 (0.47, 6.80)	0.024	−22.56 (-25.73 to −19.39)	< 0.001	−26.12 (-29.28 to −22.95)	< 0.001	−24.93 (-28.1 to −21.77)	<0.001
Difference from heparin-free (95% CI)^b^	28.57 (24.82 to 32.32)	< 0.001	2.37 (-1.38 to 6.12)	0.215	−1.18 (-4.93 to 2.57)	0.536	Ref.	
**Substitute rate**								
Predilution	174.89 ± 17.51		170.55 ± 16.92		182.11 ± 25.38		182.44 ± 26.13	0.181^c^
Postdilution	190.04 ± 23.20		76.55 ± 10.06		73.28 ± 11.98		78.55 ± 8.98	< 0.001^c^
Change from pre to post (95% CI)^a^	15.15 (1.96 to 28.35)	0.024	−94.01 (-107.20 to −80.81)	< 0.001	−108.83 (-122.02 to −95.63)	< 0.001	−103.89 (-117.09 to −90.70)	<0.001
Difference from heparin-free (95% CI)^b^	119.05 (103.42 to 134.68)	< 0.001	9.89 (-5.74 to 25.52)	0.215	−4.93 (-20.56 to 10.7)	0.536	Ref.	
**Ultrafiltration**								
Predilution	2.64 ± 0.98		2.65 ± 0.91		2.46 ± 0.80		2.56 ± 1.08	0.709^c^
Postdilution	2.15 ± 0.84		2.35 ± 1.02		2.29 ± 1.07		2.35 ± 0.94	0.701^c^
Change from pre to post (95% CI)^a^	−0.49 (−0.91 to −0.06)	0.024	−0.30 (−0.73 to 0.12)	0.163	−0.17 (−0.59 to 0.25)	0.431	−0.20 (−0.63 to 0.22)	0.350
Difference from heparin-free (95% CI)^b^	−0.29 (−0.77 to 0.20)	0.251	−0.10 (−0.59 to 0.39)	0.689	0.03 (−0.46 to 0.52)	0.898	Ref.	
**Effective convective volume**								
Predilution	22.33 ± 1.08		22.65 ± 0.96		23.29 ± 1.57		23.23 ± 1.46	0.087^c^
Postdilution	23.41 ± 1.59		20.72 ± 1.58		19.87 ± 2.45		21.21 ± 1.75	< 0.001^c^
Change from predilution (95% CI)^a^	1.08 (−0.04 to 2.20)	0.059	−1.93 (-3.05 to −0.81)	0.001	−3.41 (-4.54 to −2.29)	< 0.001	−2.02 (-3.15 to −0.90)	< 0.001
Difference from heparin-free (95% CI)^b^	3.11 (1.63 to 4.58)	<0.001	0.10 (-1.38 to 1.57)	0.899	−1.39 (-2.87 to 0.09)	0.065	Ref.	
**Hct**								
Predilution	35.23 ± 4.47		32.54 ± 3.95		32.39 ± 4.44		33.28 ± 4.01	0.119^c^
Postdilution	33.33 ± 4.30		34.26 ± 3.20		32.26 ± 2.74		32.76 ± 3.37	0.603^c^
Change from pre to post (95% CI)^a^	−1.90 (-4.68 to 0.88)	0.181	1.73 (-1.06 to 4.51)	0.225	−0.13 (-2.91 to 2.66)	0.930	−0.51 (-3.30 to 2.27)	0.718
Difference from heparin-free (95% CI)^b^	−1.39 (-4.96 to 2.19)	0.447	2.24 (-1.34 to 5.81)	0.220	0.39 (-3.19 to 3.96)	0.832	Ref.	
**DF**								
Predilution	50.18 ± 2.35		52.13 ± 2.85		50.8 ± 3.75		50.55 ± 4.27	0.238^c^
Postdilution	49.24 ± 2.94		–		–		–	–
Change from predilution (95% CI)^a^	−0.94 (-2.91 to 1.04)	0.352	–	–	–	–	–	–
Difference from heparin-free (95% CI)^b^	–	–	–	–	–	–	Ref.	
**Qfeff**								
Predilution	87.46 ± 5.50		88.54 ± 5.13		91.77 ± 6.81		91.31 ± 6.48	0.051^c^
Postdilution	93.07 ± 6.62		76.55 ± 10.06		73.28 ± 11.98		78.55 ± 8.98	< 0.001^c^
Change from predilution (95% CI)^a^	5.62 (0.43 to 10.80)	0.034	−11.99 (-17.18 to −6.80)	< 0.001	−18.49 (-23.68 to −13.30)	< 0.001	−12.76 (-17.95 to −7.57)	< 0.001
Difference from heparin-free (95% CI)^b^	18.38 (11.43 to 25.33)	< 0.001	0.77 (-6.18 to 7.72)	0.827	−5.72 (-12.68 to 1.23)	0.106	Ref.	

^a^ Absolute difference is the mean change from pre-dilution with 95% CIs in the same phase estimated by a linear mixed-effects model adjusted for baseline.

^b^ Absolute difference is the mean difference of each parameter from difference in heparin free with 95% Cis in the same phase estimated by a linear mixed-effects model adjusted for baseline.

^c^ Different time point estimated by One Way repeated measures ANOVA.

Significant if *p* < 0.05.

DF: Dilution factor; Qfeff: effective convection flow rate = substitute volume*1000/t*DF.

### Clotting scores

The mean clotting scores in the pre-dilution mode were significantly lower than those in the post-dilution mode in all phases, except in the heparin-free phase (*p* = 0.001 in phase 1 and *p* = 0.023 in phase 2). The clotting scores were 1.65 ± 0.27 in the pre-dilution baseline phase and increased to 1.88 ± 0.41 in phase 1, 2.10 ± 0.233 in phase 2, and 2.48 ± 0.27 in phase 3 (*p* < 0.01). The clotting scores in the post-dilution phases were 2.19 ± 0.46 at baseline, 2.32 ± 0.17 in phase 1, 2.42 ± 0.23 in phase 2, and 2.51 ± 0.18) in phase 3 (*p* = 0.167) (Table 4). Although the clotting score progressively increased from phase 1 to phase 3, and the number of dialyzer reuses significantly increased both in the pre- and post-dilution phases in the heparin-free phase (*p* < 0.001), the maximum scores were less than 25%, which can be reused effectively with acceptable adequacy of dialysis.

**Table 4.** Clotting scores in various phases in pre and postdilution oL-HDF.

Visual clotting score	Predilution	Postdilution	Difference (95% CI)^a^	*p* value
pre/washout	1.65 ± 0.27	2.19 ± 0.46	0.54 (0.27, 0.81)	<0.001*
Phase 1 (heparin 50%)	1.88 ± 0.41	2.32 ± 0.17	0.45 (0.18, 0.72)	0.001*
Phase 2 (heparin 25%)	2.10 ± 0.23	2.42 ± 0.23	0.31 (0.04, 0.58)	0.023
Phase 3 (no heparin)	2.48 ± 0.27	2.51 ± 0.18	0.03 (−0.24, 0.30)	0.815

Absolute difference is the mean change from pre -dilution in the same phase with 95% CIs estimated by a linear mixed-effects model adjusted for baseline.

### Adequacy of dialysis

The values of Kt/V in both modalities were comparable except for the baseline phase, in which the values of pre-dilution were significantly greater than post-dilution mode (2.36 ± 0.52 vs. 1.87 ± 0.33;95% CI −0.81 to −0.19, *p* = 0.002) (Table 5). The Kt/V in heparin anticoagulation (baseline phase) was significantly higher than that in citrate anticoagulation in the predilution mode (*p* = 0.006), while there was no significant difference in the adequacy of dialysis in the postdilution mode between heparin and citrate anticoagulation (*p* = 0.610).

**Table 5.** Biochemical parameters, ESA doses, iron supplement per week, and reuse number of dialyzers after changing from acetate to citrate dialysate and reduce heparin in stepwise fashion.

Labs								
Outcome	Pre phase		Phase 1 heparin 50%		Phase 2 heparin 25%		Phase 3 heparin-free	
	Mean ± SD/Absolute difference	*p* Value	Mean ± SD/Absolute difference	*p* Value	Mean ± SD/Absolute difference	*p* Value	Mean ± SD/Absolute difference	*p* Value
Hemoglobin								
Pre-dilution	11.20 ± 1.21		10.38 ± 1.05		10.29 ± 1.16		10.74 ± 1.08	0.168^c^
Post-dilution	10.68 ± 1.26		10.86 ± 0.82		10.26 ± 0.95		10.49 ± 0.92	0.659^c^
Change from pre-dilution (95% CI)^a^	−0.53 (-1.41 to 0.36)	0.247	0.49 (−0.40 to 1.38)	0.282	−0.03 (−0.91 to 0.86)	0.956	−0.25 (-1.14 to 0.64)	0.581
Difference from heparin-free (95% CI)^b^	−0.28 (-1.43 to 0.88)	0.642	0.74 (−0.42 to 1.90)	0.212	0.23 (−0.93 to 1.38)	0.704	Ref.	
ESA Dose (%)								
Predilution	100.00 ± 0		100.00 ± 46.29		104.75 ± 42.20		104.13 ± 29.16	0.842^c^
Postdilution	96.88 ± 20.86		106.25 ± 17.68		106.88 ± 18.34		125.00 ± 38.87	0.189^c^
Change from pre-dilution (95% CI)^a^	−3.13 (-30.3 to 24.05)	0.822	6.25 (-20.92 to 33.42)	0.652	2.125 (-25.05 to 29.3)	0.878	20.88 (-6.3 to 48.05)	0.132
Difference from heparin-free (95% CI)^b^	−24 (-53.92 to 5.92)	0.116	−14.63 (-44.54 to 15.29)	0.338	−18.75 (-48.67 to 11.17)	0.219	Ref.	
Ferritin								
Predilution	506.34 ± 446.70		601.48 ± 491.20		803.25 ± 461.82		611.88 ± 275.42	0.090^c^
Postdilution	513.75 ± 326.29		916.50 ± 748.54		545.91 ± 317.19		449.50 ± 291.82	0.110^c^
Change from pre-dilution (95% CI)^a^	7.41 (-294.75 to 309.57)	0.962	315.03 (12.87 to 617.18)	0.041	−257.34 (-559.5 to 44.82)	0.095	−162.38 (-464.53 to 139.78)	0.292
Difference from heparin-free (95% CI)^b^	169.79 (-200.98, 540.55)	0.369	477.40 (106.64 to 848.16)	0.012	−94.96 (-465.73 to 275.80)	0.616	Ref.	
Iron supplement per wk								
Predilution	25.00 ± 26.73		28.13 ± 36.44		75.00 ± 90.63		28.13 ± 41.05	0.216^c^
Postdliution	31.25 ± 70.39		43.75 ± 87.37		12.5 ± 26.73		21.88 ± 24.78	0.636^c^
Change from pre-dilution (95% CI)^a^	6.25 (-43.91 to 56.41)	0.807	15.63 (-34.53 to 65.78)	0.541	−62.50 (-112.66 to −12.34)	0.015	−6.25 (-56.41 to 43.91)	0.807
Difference from heparin-free (95% CI)^b^	12.5 (-58.43 to 83.43)	0.730	21.88 (-49.06 to 92.81)	0.546	−56.25 (-127.18 to 14.68)	0.120	Ref.	
Tsat								
Predilution	0.32 ± 0.10		0.24 ± 0.07		0.31 ± 0.09		0.33 ± 0.11	0.088^c^
Postdilution	0.30 ± 0.10		0.34 ± 0.08		0.30 ± 0.10		0.26 ± 0.07	0.276^c^
Change from pre-dilution (95% CI)^a^	−0.02 (−0.09 to 0.05)	0.618	0.10 (0.03 to 0.17)	0.004	−0.01 (−0.08 to 0.05)	0.667	−0.07 (−0.14 to 0)	0.053
Difference from heparin-free (95% CI)^b^	0.05 (−0.04 to 0.14)	0.297	0.17 (0.07 to 0.26)	< 0.001	0.05 (−0.04 to 0.15)	0.274	Ref.	
Kt/V								
Predilution	2.36 ± 0.52		1.74 ± 0.49		1.98 ± 0.28		1.91 ± 0.37	0.006^c^
Postdilution	1.87 ± 0.33		1.91 ± 0.38		2.00 ± 0.62		1.83 ± 0.36	0.610^c^
Change from pre-dilution (95% CI)^a^	−0.50 (−0.81 to −0.19)	0.002	0.17 (−0.14 to 0.48)	0.286	0.02 (−0.29 to 0.33)	0.900	−0.08 (−0.39 to 0.23)	0.627
Difference from heparin-free (95% CI)^b^	−0.42 (−0.83 to −0.01)	0.046	0.25 (−0.17 to 0.66)	0.241	0.10 (−0.32 to 0.51)	0.644	Ref.	
Ca								
Predilution	9.23 ± 0.88		8.85 ± 0.72		8.56 ± 0.66		8.54 ± 0.75	0.022^c^
Postdilution	8.79 ± 0.76		8.41 ± 1.17		8.25 ± 0.83		8.50 ± 0.76	0.230^c^
Change from pre-dilution (95% CI)^a^	−0.44 (−0.94 to 0.06)	0.086	−0.44 (−0.94 to 0.06)	0.086	−0.31 (−0.81 to 0.19)	0.220	−0.04 (−0.54 to 0.46)	0.883
Difference from heparin-free (95% CI)^b^	−0.40 (-1.04 to 0.24)	0.220	−0.40 (-1.04 to 0.24)	0.220	−0.28 (−0.91 to 0.36)	0.399	Ref.	
iCa								
Predilution	4.65 ± 0.34		4.46 ± 0.19		4.45 ± 0.30		4.59 ± 0.29	0.180^c^
Postdilution	4.56 ± 0.29		4.56 ± 0.37		4.51 ± 0.46		4.67 ± 0.21	0.331^c^
Change from pre-diution (95% CI)^a^	−0.08 (−0.30 to 0.13)	0.451	0.09 (−0.12 to 0.31)	0.391	0.06 (−0.15 to 0.27)	0.583	0.08 (−0.14 to 0.29)	0.472
Difference from heparin-free (95% CI)^b^	−0.16 (−0.41 to 0.09)	0.206	0.02 (−0.23 to 0.26)	0.906	−0.02 (−0.27 to 0.23)	0.883	Ref.	
iPTH								
Predilution	406.49 ± 307.70		553.55 ± 435.21		524.84 ± 351.41		494.74 ± 292.65	0.665^c^
Postdilution	421.29 ± 267.77		377.78 ± 199.09		345.81 ± 267.12		304.90 ± 195.91	0.517^c^
Change from pre-dilution (95% CI)^a^	14.80 (-192.23 to 221.83)	0.889	−175.78 (-382.80 to 31.25)	0.096	−179.03 (-386.05 to 28.00)	0.090	−189.84 (-396.87 to 17.19)	0.072
Difference from heparin-free (95% CI)^b^	204.64 (-62.58 to 471.85)	0.133	14.06 (-253.15 to 281.28)	0.918	10.81 (-256.40 to 278.03)	0.937	Ref.	
hs-CRP								
Predilution	4.19 ± 4.53		7.24 ± 10.54		6.30 ± 9.43		6.04 ± 5.42	0.810^c^
Postdilution	4.87 ± 5.05		4.76 ± 3.47		9.70 ± 12.86		5.21 ± 3.89	0.342^c^
Change from pre -dilution (95% CI)^a^	0.68 (-5.09 to 6.45)	0.817	−2.90 (-8.86 to 3.07)	0.341	2.99 (-2.98 to 8.96)	0.326	−1.25 (-7.22 to 4.72)	0.681
Difference from heparin-free (95% CI)^b^	1.93 (-5.80 to 9.66)	0.624	−1.65 (-9.49 to 6.20)	0.681	4.24 (-3.60 to 12.09)	0.289	Ref.	
IL-6								
Predilution	6.22 ± 3.68		7.03 ± 5.23		7.49 ± 4.55		5.94 ± 3.99	0.316^c^
Postdilution	6.45 ± 4.07		6.99 ± 3.19		6.73 ± 3.79		7.32 ± 3.70	0.869^c^
Change from pre -dilution (95% CI)^a^	0.23 (-2.02 to 2.48)	0.840	−0.45 (-2.86 to 1.96)	0.716	−0.88 (-3.32 to 1.56)	0.478	1.13 (-1.20 to 3.47)	0.342
Difference from heparin-free (95% CI)^b^	−0.90 (-3.93 to 2.13)	0.559	−1.58 (-4.70 to 1.54)	0.321	−2.02 (-5.16 to 1.13)	0.209	Ref.	
max use								
Predilution	6.00 ± 0		9.38 ± 2.88		6.50 ± 3.63		7.75 ± 2.92	0.050^c^
Postdilution	5.25 ± 1.04		8.00 ± 2.27		7.25 ± 2.82		5.63 ± 1.92	0.014^c^
Change from pre-dilution (95% CI)^a^	−0.75 (-2.77 to 1.27)	0.466	−1.38 (-3.39 to 0.64)	0.182	0.75 (-1.27 to 2.77)	0.466	−2.13 (-4.14 to −0.11)	0.039
Difference from heparin-free (95% CI)^b^	1.38 (-1.31 to 4.06)	0.315	0.75 (-1.93 to 3.43)	0.584	2.88 (0.19 to 5.56)	0.036		

^a^ Absolute difference is the mean change from pre -dilution in the same phase with 95% CIs estimated by a linear mixed-effects model adjusted for baseline.

^b^ Absolute difference is the mean difference of each parameter from difference in heparin-free with 95% Cis in the same phase estimated by a linear mixed-effects model adjusted for baseline.

^c^ Different time point estimated by One-Way repeated measures ANOVA.

Significant if *p* < 0.05.

Max use: Number of reuses in each phase.

iCa: ionized calcium.

### Clinical and laboratory parameters

The laboratory evaluations in the two treatment periods are shown in Table 5, together with the adequacy of dialysis and ESA dosages.

### Effect on anemia

**Figure 2.**
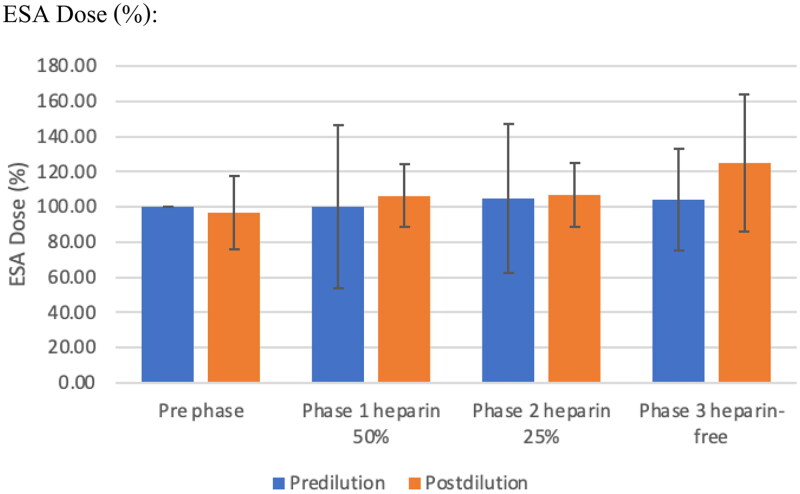
Comparison between erythrocyte stimulating agents dose between pre-and post- dilution online hemodiafiltration.

The mean hemoglobin and ESA doses were not significantly different in each and between pre- and post-protocol (Figure 2). The median dose of ESA was not significantly different among the three study periods. Transferrin saturation (TSAT) declined from baseline to phase 1 in the pre-dilution mode (*p* = 0.004), but was not significantly different in the post-dilution mode. Serum ferritin increased progressively in the pre-dilution mode from baseline throughout Phase 2 and dropped in Phase 3 (601.48 ± 491, 20,803.25 ± 461.82, 611.88 ± 275 ng/mL in phases 1, 2, and 3, respectively, *p* = 090). The mean difference values of serum ferritin in phase 1 were significantly higher than in baseline in the pre-dilution mode (*p* = 0.041), and the values in the post-HDF group were significantly higher than in the pre-dilution mode in phase 1 (601.48 ± 491.20 ng/mL vs. 916.50 ± 748.54 ng/mL, *p* = 0.012). This corresponds to the administration of intravenous iron in six patients in the pre-dilution mode and five in the post-dilution mode (Table 5)

### Calcium and phosphate balance and acid-base status

The baseline pre- and post-dilution levels of total serum calcium, ionized calcium, and iPTH were not significantly different between phases. The phosphorus levels in the post-dilution phase 1 were lower than in the pre-dilution mode and in the heparin-free phase (-2.05, 95% confidence interval [CI] − 3.12 to −0.98 absolute difference between the pre- and post-dilution in phase 1, *p* < 0.001 and −2.23, 95% CI −3.71 to −0.74 absolute difference compared to phase 3, *p* = 0.003 (Table 5).

### Inflammatory cytokines

In both modes, there was no significant change in the levels of inflammatory cytokines (high-sensitivity C-reactive protein [hs-CRP] and interleukin [IL]-6) or across the HDF protocol or heparin phase, indicating no activation of the inflammatory cascade (Table 5).

### Quality of life

**Figure 3.**
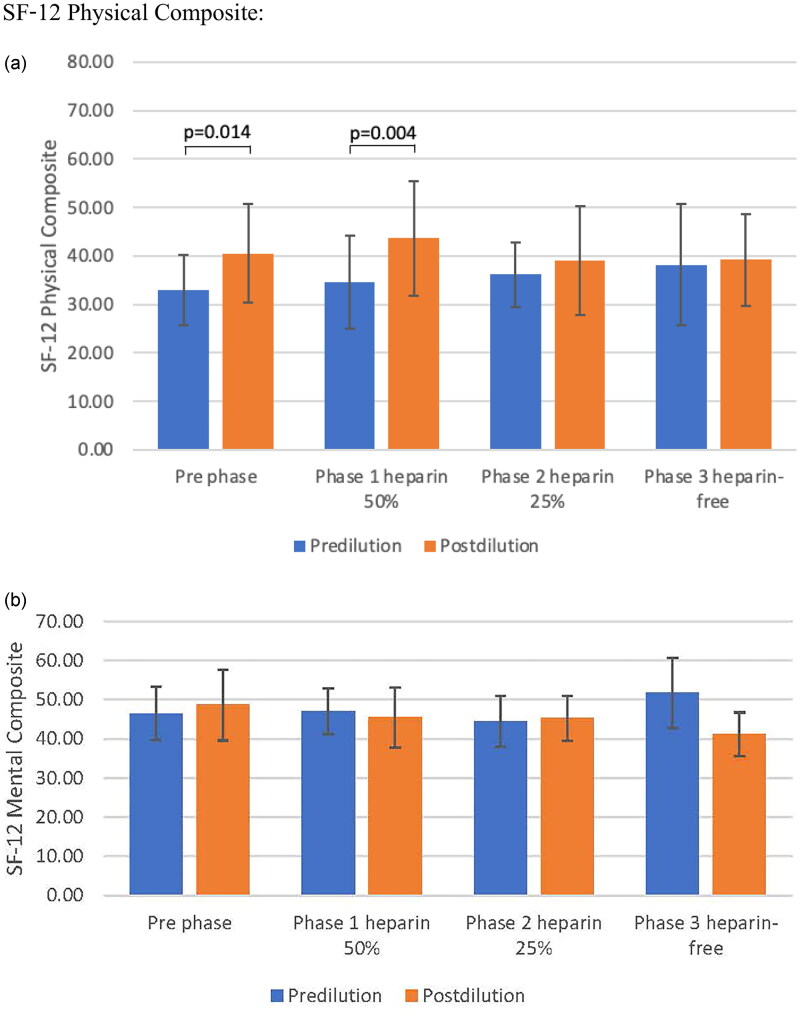
Mental component summary (MCS) and physical component summary (PCS) are comparable between pre and post-dilution mode A: Physical component B: Mental component Comparison between erythrocyte stimulating agents between pre- and post- dilution online hemodiafiltration

According to the KDQOL_SF v 1.3 scoring system, the mean scores of the items related to physical functioning (PCS) of the total study population were significantly higher in the post-dilution mode than in the pre-dilution mode both at baseline and phase 1 (*p* = 0.014 and 0.004 at baseline and phase 1, respectively). The mean scores of the KDQOL-SF-12 were not far from the mean score of 50, as mentioned previously [22]. The mental component summary (MCS) was comparable between the pre- and post-dilution modes but declined significantly in phase 3 of the post-dilution mode compared with baseline (*p* = 0.006 in phase 1 and *p* < 0.001 in phase 2) (Figure 3).

For subdomain scores, the mean of the scores of the item related to the ‘symptoms and problem’ list of the post-dilution at baseline was 82.29 ± 11.99 and 84.64 ± 9.89 in phase 1, respectively, which was nearly the maximum possible score of 100 for the least symptoms and problems. The mean scores in the post-dilution mode were greater than in the pre-dilution mode in this item as well as other items such as ‘burden of kidney diseases’, quality of social interaction’, ‘sleep’, ‘social interaction’, ‘emotional well-being’, ‘social-function’, ‘physical functioning’, and ‘energy’ except for ‘cognitive function’, in which the pre-dilution mode was greater in the post-dilution mode. On the other hand, the mean scores of the items dealing with ‘work status’, ‘physical function’, and ‘burden of kidney disease’ were very low (31.25 ± 25.88, 25.00 ± 29.28, and 6.25 ± 11.57, respectively, at baseline), indicating high age in this group of patients and poor physical function at baseline (Table 6).

**Table 6.** KDQOL-SF-12 physical /mental composite score.

Outcome	Pre phase		Phase 1 heparin 50%		Phase 2 heparin 25%		Phase 3 heparin-free	
	Mean ± SD/Absolute difference	*p* Value	Mean ± SD/Absolute difference	*p* Value	Mean ± SD/Absolute difference	*p* Value	Mean ± SD/Absolute difference	*p* Value
SF-12 Physical Composite								
Predilution	32.96 ± 7.21		34.67 ± 9.56		36.14 ± 6.66		38.21 ± 12.52	
Postdilution	40.56 ± 10.17		43.64 ± 11.77		38.96 ± 11.25		39.25 ± 9.51	
Change from predilution (95% CI)^a^	7.6 (1.57, 13.63)	0.014	8.97 (2.94, 15.00)	0.004	2.82 (-3.21, 8.85)	0.359	1.03 (-4.99, 7.06)	0.737
Difference from heparin-free (95% CI)^b^	6.56 (-1.24, 14.36)	0.099	7.94 (0.13, 15.74)	0.046	1.79 (-6.01, 9.59)	0.653	Ref.	
SF-12 Mental Composite								
Predilution	46.47 ± 6.79		47.14 ± 5.86		44.52 ± 6.46		51.73 ± 8.92	
Postdilution	48.70 ± 9.08		45.48 ± 7.74		45.24 ± 5.83		41.21 ± 5.55	
Change from pre prodilution (95% CI)^a^	2.23 (-2.79, 7.26)	0.383	−1.66 (-6.68, 3.36)	0.517	0.72 (-4.3, 5.74)	0.780	−10.52 (-15.54, −5.5)	<0.001
Difference from heparin-free (95% CI)^b^	12.75 (6.45, 19.06)	<0.001	8.86 (2.55, 15.17)	0.006	11.24 (4.93, 17.54)	<0.001	Ref.	

^a^ Absolute difference is the mean change from predilution with 95%CIs estimated by a linear mixed-effects model adjusted for baseline.

^b^ Absolute difference is the mean difference from heparin-free with 95%CIs estimated by a linear mixed-effects model adjusted for baseline.

Significant if *p* < 0.05.

## Discussion

Recent evidence shows that the clinical benefits of HDF are directly correlated to the excellent combination of diffusive solute transport and the high clearances for larger uremic toxins, the so-called middle molecules. The advantage of this combination is that HDF is superior to conventional HD with respect to uremic toxin removal [23].

However, the main limiting factor for HDF is clotting in the extracorporeal circuit.

The post-dilution mode, that is, the substitution fluid administered after the dialyzer, increases the clearance compared with HD for both small and large uremic toxins. A limitation of post-HDF is the hemoconcentration between the dialyzer and the venous drip chamber. In the predilution mode, that is, replacement before the dialyzer will have plasma dilution, which may affect the overall efficiency of the clearance, while clotting is less than post-dilution.

Normally, unfractionated heparin and low-molecular-weight heparin are used as anticoagulants. However, anticoagulant use is limited by the risk of bleeding. Another technique to limit thrombosis in the extracorporeal circuit is regional citrate anticoagulation or CD, which has shown promising results in previous studies [24,25].

In this study, pre-dilution had less extracorporeal circuit clotting than post-dilution mode at baseline through phase 2. This phenomenon was not surprising, as it corresponds to the characteristics of pre-versus post-HDF [26,27]. The clotting scores significantly increased in the post-dilution mode compared to the pre- dilution mode, with a maximum value of less than 3. We previously reported that citrate eliminates the need for heparin during HD [25] but does not prolong the circuit life span in continuous venovenous hemodiafiltration in the post-dilution mode [24]. Previous studies have suggested that some clotting occurs during HD or continuous renal replacement therapy and that citrate can attenuate the need for heparin [28,29] but not abolish it entirely. One study from pediatric cohorts under pre-HDF divided into acetate and citrate phases observed no difference in clotting episodes and clearance between the periods [30]. Aniort et al. safely used CD post-HDF and were able to remove heparin in most patients [17]. Richtrova et al. performed a study using citrate in pre-HDF to successfully eliminate heparin. Dialysis adequacy declined significantly in the citrate group compared with the acetate group [31]. In our study, a citrate-based dialysis solution enabled heparin dose tapering or even complete exclusion, both in pre- and post-HDF, without compromising the adequacy of dialysis as quantified by spKt/V. The spKt/V in post-dilution was not significantly different between the pre- and post-dilution modes. The effective convective volume in our study was significantly higher during pre-dilution. Notably, citrate can be feasibly used both pre- and post-HDF. However, at 300 mL/min, blood flow, as performed in this study, the pre-dilution mode would lead to less thrombogenesis. Special attention should be paid to the heparin-free phase because the number of maximum reused dialyzers increased in that phase, especially in the post-dilution mode.

Previous reports have also indicated that citrate reduced iCa levels at the end of the session [15, 32]. In the study by Ortiz et al. citrate produced less post-dialysis alkalemia, significantly modified Ca, Mg, and PTH levels, and had a positive impact on hemodynamic tolerance in patients on maintenance HD. Our study did not find any electrolyte abnormalities or calcium phosphate derangements during the oL-HDF with citrate. The ionized calcium levels were stable throughout the study period. The anticoagulant effect of citrate in this study, despite the stable iCa level, may be due to its adequate anticoagulant effect. The stable iCa2 level in this study may be due to the difference in the diffusible calcium gradient (free calcium + calcium chelated by citrate), which allows calcium to be transferred from the dialysate to the patient. Citrate-chelated calcium is released during the metabolism of citrate to bicarbonate in the liver [25].

Considering the effect of citrate on anemia, stability of plasma hemoglobin and a decrease in the erythropoietin resistance index were found in a recent report [17]. We previously showed that citrate dialysate stabilized the mean hemoglobin levels during different heparin reduction phases in conventional HD [25]. The iron status was also maintained, except in post-dilution phase 1, which corresponds to iron supplements in this group of patients at that time. Our findings provided some evidence that although heparin reduction during CD was associated with a slight increase in the clotting score, hemoglobin levels were maintained, and no effects of CD on anemia were found. This could be explained by adequate iron storage and the adjustment of ESA doses according to the Hb levels of the patients to maintain Hb within acceptable ranges.

Our data also showed that CD produced similar levels of inflammatory markers as acetate. IL-6 and hs-CRP levels were not elevated during any phase of CD. This is in accordance with previous reports by Nunez et al. who reported lower inflammatory parameters, that is, CRP and β_2-_MG using CD [33]. These results are not attributable to dialysis technology (oL-HDF) and may suggest a potential biological effect of citrate on the CKD-associated inflammatory state [34].

The goal of RRT in patients with ESKD is not only to improve patient survival but also to achieve well-being [35]. This study evaluated physical, emotional, and social functions, and treatment effectiveness using multidimensional measurements, including generic and disease-specific instruments. [36]. The physical domain includes three subscales, namely, physical function, role limitation due to physical function, and bodily pain. In previous studies, this domain has been reported to be a significant predictor of both death and hospitalization [37]. In the current study, each of the three subscales of the physical domain had a score below an average of 50 in the predilution mode, which was inferior to post-dilution, suggesting that post-HDF itself has an impact on performance. This is in agreement with the higher kT/V values in post-dilution than in pre-dilution, although the difference was not statistically significant in phase 1–3. This finding emphasizes the positive impact of dialysis adequacy on QOL. Hasan et al. reported a positive association between the adequacy of dialysis using Kt/V and health-related quality of life (HRQOL) [38], while some showed no significant association [39]. On the other hand, the mental domain includes social function and role limitation due to mental and general mental health being better in the post-dilution mode, especially at baseline and Phase 1. The scores for almost all the subscales in the present study were above 50. The sleep scores were also better post-dilution, which may be the consequence of the better adequacy of dialysis in that mode.

Although overall health was not different between the two treatment groups, patient satisfaction was greater after post-dilution. CD had no effect on the physical domain, except for the role-limit subscale, in which the scores dropped in the heparin-free phase. The mental domain sub-scores declined progressively as heparin was curtailed. The patients denied answering sex-related questions. This may be due to embarrassment in traditional Thai culture and the high mean age of patients.

This trial has many strengths, including a comparison between pre- and post-dilution, which has rarely been reported previously. Additionally, an assumption to avoid heparin use was developed for both modalities. We designed the trial as a step-down of the heparin dose in a gradual framework. We explored the potential impact of dialysis modalities for CD on different parameters, including laboratory data, adequacy of dialysis, and QOL. Another advantage is the paired comparisons of various variables between both modalities in every step of heparin reduction, which could envision the benefits of improving the selection of the appropriate doses of heparin.

Our study has some limitations. First, we recruited a limited number of patients for the study. Second, the duration of follow-up was only three months, and long-term clinical and laboratory results were lacking. This study did not use a randomized-controlled or crossover design, and the cumulative effect of the pre-dilution oL-HDF on parameters such as quality of life cannot be ruled out. Further well-designed studies with a greater number of participants and longer follow-up periods are required to establish the beneficial effects of CD on oL-HDF.

In conclusion, citrate can be safely used both pre- and post-HDF. In patients with a limited blood flow rate of < 300 mL/min, the pre-dilution mode with CD is recommended. Moreover, heparin can be avoided without excessive clotting and yields comparable adequacy of dialysis, as well as maintaining calcium-phosphorus metabolism and hematologic profile. The post-dilution QOL was significantly better than the pre-dilution QOL in the physical domain. Overall health was not affected by CD or heparin reduction. Therefore, CD could be an appropriate alternative anticoagulant for oL-HDF in patients on chronic HD.

## Supplementary Material

Supplemental Material

## Data Availability

Data supporting the findings of this study are available from the corresponding author. Data supporting the findings of this study are openly available in ‘figshare’ at http://10.6084/m9.figshare.21971570.
