# Supplementary material for: Comparison of citrate dialysate in pre- and post-dilution online hemodiafiltration: effect on clot formation and adequacy of dialysis in hemodialysis patients
Source: Ren Fail. 2024 Jan 8;46(1):2302109. doi: 10.1080/0886022X.2024.2302109 (PMC10776067; doi:10.1080/0886022X.2024.2302109)
Supplement: Supplemental Material [file IRNF_A_2302109_SM2751.pdf]

KDQOL-SF-12

Outcome	Pre phase		Phase 1 heparin 50%		Phase 2 heparin 25%		Phase 3 heparin-free	
	Mean ± SD/ Absolute difference	p-value	Mean ± SD/ Absolute difference	p-value	Mean ± SD/ Absolute difference	p-value	Mean ± SD/ Absolute difference	p-value
**D1: Symptom/problem list**								
Pre dilution	70.31 ± 14.85		74.48 ± 13.72		79.95 ± 4.85		80.47 ± 16.29	0.230[c]
Post dilution	82.29 ± 11.99		84.64 ± 9.89		78.39 ± 12.89		77.34 ± 11.43	0.065[c]
Change from pre dilution (95% CI)[a]	11.98 (2.56 , 21.4)	0.013	10.16 (0.73 , 19.58)	0.035	-1.56 (-10.98 , 7.86)	0.745	-3.13 (-12.55 , 6.30)	0.516
Difference from heparin-free (95% CI)[b]	15.10 (3.87 , 26.34)	0.008	13.28 (2.04 , 24.52)	0.021	1.56 (-9.67 , 12.80)	0.785	Ref.	
**D2: Effects of kidney disease**								
Pre dilution	62.50 ± 19.62		64.84 ± 15.19		68.36 ± 13.09		67.58 ± 17.51	0.555[c]
Post dilution	63.67 ± 25.22		63.67 ± 23.38		66.80 ± 17.83		58.98 ± 26.85	0.725[c]
Change from pre dilution (95% CI)[a]	1.17 (-13.90 , 16.25)	0.879	-1.17 (-16.25 , 13.90)	0.879	-1.56 (-16.64 , 13.51)	0.839	-8.59 (-23.67 , 6.48)	0.264
Difference from heparin-free (95% CI)[b]	9.77 (-9.11 , 28.64)	0.311	7.42 (-11.45 , 26.30)	0.441	7.03 (-11.84 , 25.91)	0.465	Ref.	
**D3: Burden of kidney disease**								
Pre dilution	6.25 ± 11.57		7.03 ± 9.11		16.41 ± 23.13		46.88 ± 25.88	0.008[c]
Post dilution	45.31 ± 20.25		30.47 ± 24.87		27.34 ± 21.38		20.31 ± 14.47	0.007[c]
Change from pre dilution (95% CI)[a]	39.06 (23.11 , 55.02)	<0.001	23.44 (7.48 , 39.39)	0.004	10.94 (-5.02 , 26.89)	0.179	-26.56 (-42.52 , -10.61)	0.001
Difference from heparin-free (95% CI)[b]	65.63 (43.85 , 87.4)	<0.001	50 (28.23 , 71.77)	<0.001	37.5 (15.73 , 59.27)	0.001	Ref.	
**D4: Work status**								
Pre dilution	31.25 ± 25.88		31.25 ± 25.88		31.25 ± 25.88		50.00 ± 0	0.080[c]
Post dilution	37.50 ± 23.15		43.75 ± 17.68		50.00 ± 26.73		56.25 ± 17.68	0.170[c]
Change from pre dilution (95% CI)[a]	6.25 (-9.02 , 21.52)	0.422	12.5 (-2.77 , 27.77)	0.109	18.75 (3.48 , 34.02)	0.016	6.25 (-9.02 , 21.52)	0.422
Difference from heparin-free (95% CI)[b]	0 (-20.29 , 20.29)	1.000	6.25 (-14.04 , 26.54)	0.546	12.5 (-7.79 , 32.79)	0.227	Ref.	
**D5: Cognitive function**								
Pre dilution	75.00 ± 7.77		77.50 ± 7.07		71.67 ± 6.90		63.33 ± 9.43	0.003[c]
Post dilution	65.83 ± 9.04		68.33 ± 11.13		65.00 ± 17.00		63.33 ± 10.08	0.845[c]

Information Classification: General

Change from pre dilution (95% CI)[a]	-9.17 (-18.07 , -0.27)	0.043	-9.17 (-18.07 , -0.27)	0.043	-6.67 (-15.57 , 2.23)	0.142	0 (-8.90 , 8.90)	1.000
Difference from heparin-free (95% CI)[b]	-9.17 (-21.63 , 3.30)	0.149	-9.17 (-21.63 , 3.30)	0.149	-6.67 (-19.13 , 5.80)	0.295	Ref.	
**D6: Quality of social interaction**								
Pre dilution	66.67 ± 11.82		67.50 ± 10.95		70.00 ± 12.34		81.67 ± 16.62	0.057[c]
Post dilution	75.00 ± 24.10		75.00 ± 15.43		65.83 ± 20.91		64.17 ± 20.14	0.299[c]
Change from pre dilution (95% CI)[a]	8.33 (-6.11 , 22.78)	0.258	7.50 (-6.94 , 21.94)	0.309	-4.17 (-18.61 , 10.28)	0.572	-17.50 (-31.94 , -3.06)	0.018
Difference from heparin-free (95% CI)[b]	25.83 (8.80 , 42.87)	0.003	25.00 (7.96 , 42.04)	0.004	13.33 (-3.70 , 30.37)	0.125	Ref.	
**D7: Sexual function**								
Pre dilution	-		-		-		-	-
Post dilution	-		-		-		-	-
Change from pre dilution (95% CI)[a]	-	-	-	-	-	-	-	-
Difference from heparin-free (95% CI)[b]	-	-	-	-	-	-	Ref.	-
**D8: Sleep**								
Pre dilution	55.00 ± 3.78		58.44 ± 7.90		59.38 ± 9.61		69.38 ± 11.32	0.021[c]
Post dilution	70.00 ± 11.73		60.00 ± 12.68		57.81 ± 13.79		52.81 ± 13.66	0.021[c]
Change from pre dilution (95% CI)[a]	15.00 (6.19 , 23.81)	0.001	1.56 (-7.25 , 10.37)	0.728	-1.56 (-10.37 , 7.25)	0.728	-16.56 (-25.37 , -7.75)	<0.001
Difference from heparin-free (95% CI)[b]	31.56 (19.53 , 43.60)	<0.001	18.13 (6.09 , 30.16)	0.003	15.00 (2.97 , 27.03)	0.015	Ref.	
**D9: Social support**								
Pre dilution	62.50 ± 21.36		66.67 ± 8.91		68.75 ± 10.68		89.58 ± 15.27	0.013[c]
Post dilution	83.33 ± 25.20		85.42 ± 24.30		79.17 ± 24.80		79.17 ± 24.80	0.473[c]
Change from pre dilution (95% CI)[a]	20.83 (5.87 , 35.80)	0.006	18.75 (3.79 , 33.71)	0.014	10.42 (-4.55 , 25.38)	0.172	-10.42 (-25.38 , 4.55)	0.172
Difference from heparin-free (95% CI)[b]	31.25 (14.62 , 47.88)	<0.001	29.17 (12.53 , 45.8)	0.001	20.83 (4.2 , 37.47)	0.014	Ref.	
**D10: Dialysis staff encouragement**								
Pre dilution	73.44 ± 4.42		75.00 ± 0		84.38 ± 11.08		93.75 ± 9.45	<0.001[c]
Post dilution	81.25 ± 9.45		82.81 ± 11.45		84.38 ± 12.94		84.38 ± 12.94	0.953[c]
Change from pre dilution (95% CI)[a]	7.81 (-1.28 , 16.90)	0.092	7.81 (-1.28 , 16.90)	0.092	0 (-9.09 , 9.09)	1.000	-9.38 (-18.46 , -0.29)	0.043

Information Classification: General

Difference from heparin-free (95% CI)[b]	17.19 (4.33 , 30.04)	0.009	17.19 (4.33 , 30.04)	0.009	9.38 (-3.48 , 22.23)	0.153	Ref.	
**D11: Overall Health**								
Pre dilution	63.75 ± 10.61		68.75 ± 9.91		70.00 ± 7.56		62.5 ± 10.35	0.307[c]
Post dilution	66.25 ± 9.16		65.00 ± 14.14		62.5 ± 14.88		61.25 ± 9.91	0.639[c]
Change from pre dilution (95% CI)[a]	2.50 (-6.02 , 11.02)	0.565	-3.75 (-12.27 , 4.77)	0.388	-7.50 (-16.02 , 1.02)	0.084	-1.25 (-9.77 , 7.27)	0.774
Difference from heparin-free (95% CI)[b]	3.75 (-7.71 , 15.21)	0.521	-2.50 (-13.96 , 8.96)	0.669	-6.25 (-17.71 , 5.21)	0.285	Ref.	
**D12: Patient satisfaction**								
Pre dilution	56.25 ± 12.40		58.33 ± 12.60		56.25 ± 8.63		79.17 ± 19.42	0.040[c]
Post dilution	77.08 ± 15.27		77.08 ± 12.40		64.58 ± 22.6		68.75 ± 16.52	0.354[c]
Change from pre dilution (95% CI)[a]	20.83 (6.96 , 34.71)	0.003	18.75 (4.88 , 32.62)	0.008	8.33 (-5.54 , 22.21)	0.239	-10.42 (-24.29 , 3.46)	0.141
Difference from heparin-free (95% CI)[b]	31.25 (11.65 , 50.85)	0.002	29.17 (9.57 , 48.77)	0.004	18.75 (-0.85 , 38.35)	0.061	Ref.	
**D13: Physical functioning**								
Pre dilution	25.00 ± 29.28		26.88 ± 28.28		32.50 ± 33.38		45.00 ± 31.62	0.002[c]
Post dilution	43.75 ± 30.21		55.63 ± 42.80		50.00 ± 38.91		46.88 ± 37.41	0.653[c]
Change from pre dilution (95% CI)[a]	18.75 (-0.09 , 37.59)	0.051	28.75 (9.91 , 47.59)	0.003	17.50 (-1.34 , 36.34)	0.069	1.88 (-16.96 , 20.71)	0.845
Difference from heparin-free (95% CI)[b]	16.88 (-6.74 , 40.49)	0.161	26.88 (3.26 , 50.49)	0.026	15.63 (-7.99 , 39.24)	0.195	Ref.	
**D14: Role--physical**								
Pre dilution	31.25 ± 45.81		37.50 ± 29.88		43.75 ± 22.16		62.50 ± 46.29	0.331[c]
Post dilution	78.13 ± 33.91		59.38 ± 49.89		56.25 ± 41.73		50.00 ± 48.18	0.245[c]
Change from pre dilution (95% CI)[a]	46.88 (18.08 , 75.67)	0.001	21.88 (-6.92 , 50.67)	0.137	12.50 (-16.30 , 41.30)	0.395	-12.50 (-41.30 , 16.30)	0.395
Difference from heparin-free (95% CI)[b]	59.38 (19.77 , 98.98)	0.003	34.38 (-5.23 , 73.98)	0.089	25.00 (-14.60 , 64.60)	0.216	Ref.	
**D15: Pain**								
Pre dilution	52.81 ± 26.30		59.69 ± 21.44		62.19 ± 18.59		56.56 ± 34.38	0.759[c]
Post dilution	65.63 ± 22.59		65.94 ± 26.15		62.50 ± 24.71		64.06 ± 31.37	0.815[c]
Change from pre dilution (95% CI)[a]	12.81 (-7.18 , 32.80)	0.209	6.25 (-13.74 , 26.24)	0.540	0.31 (-19.68 , 20.30)	0.976	7.50 (-12.49 , 27.49)	0.462
Difference from heparin-free (95% CI)[b]	5.31 (-21.32 , 31.95)	0.696	-1.25 (-27.88 , 25.38)	0.927	-7.19 (-33.82 , 19.45)	0.597	Ref.	
**D16: General health**								

Information Classification: General

Pre dilution	40.63 ± 12.08		43.75 ± 14.58		43.13 ± 14.38		55.00 ± 18.71	0.246[c]
Post dilution	47.50 ± 18.71		50.63 ± 19.35		46.25 ± 15.98		39.38 ± 16.78	0.260[c]
Change from pre dilution (95% CI)[a]	6.88 (-5.88 , 19.63)	0.291	6.88 (-5.88 , 19.63)	0.291	3.13 (-9.63 , 15.88)	0.631	-15.63 (-28.38 , -2.87)	0.016
Difference from heparin-free (95% CI)[b]	22.50 (5.15 , 39.85)	0.011	22.50 (5.15 , 39.85)	0.011	18.75 (1.40 , 36.10)	0.034	Ref.	
**D17: Emotional well-being**								
Pre dilution	53.50 ± 9.78		56.50 ± 9.67		56.50 ± 7.54		68.50 ± 15.78	0.089[c]
Post dilution	64.00 ± 18.14		66.50 ± 14.65		59.50 ± 15.33		62.00 ± 11.31	0.398[c]
Change from pre dilution (95% CI)[a]	10.50 (0.92 , 20.08)	0.032	10.00 (0.42 , 19.58)	0.041	3.00 (-6.58 , 12.58)	0.540	-6.50 (-16.08 , 3.08)	0.184
Difference from heparin-free (95% CI)[b]	17.00 (4.71 , 29.29)	0.007	16.50 (4.21 , 28.79)	0.008	9.50 (-2.79 , 21.79)	0.130	Ref.	
**D18: Role--emotional**								
Pre dilution	58.33 ± 46.29		66.67 ± 25.20		58.33 ± 34.50		79.17 ± 39.59	0.557[c]
Post dilution	79.17 ± 39.59		58.33 ± 49.60		70.83 ± 41.55		50.00 ± 47.14	0.161[c]
Change from pre dilution (95% CI)[a]	20.83 (-10.84 , 52.51)	0.197	-8.33 (-40.01 , 23.34)	0.606	12.5 (-19.17 , 44.17)	0.439	-29.17 (-60.84 , 2.51)	0.071
Difference from heparin-free (95% CI)[b]	50.00 (10.19 , 89.81)	0.014	20.83 (-18.98 , 60.65)	0.305	41.67 (1.85 , 81.48)	0.040	Ref.	
**D19: Social function**								
Pre dilution	46.88 ± 18.60		53.13 ± 12.94		59.38 ± 12.94		70.31 ± 21.06	0.090[c]
Post dilution	62.50 ± 26.73		64.06 ± 18.22		60.94 ± 14.07		56.25 ± 14.94	0.418[c]
Change from pre dilution (95% CI)[a]	15.63 (2.01 , 29.24)	0.025	10.94 (-2.68 , 24.55)	0.115	1.56 (-12.05 , 15.18)	0.822	-14.06 (-27.68 , -0.45)	0.043
Difference from heparin-free (95% CI)[b]	29.69 (11.58 , 47.79)	0.001	25.00 (6.90 , 43.10)	0.007	15.63 (-2.48 , 33.73)	0.091	Ref.	
**D20: Energy/fatigue**								
Pre dilution	50.00 ± 7.07		50.63 ± 8.63		51.25 ± 6.41		68.13 ± 15.34	0.025[c]
Post dilution	61.88 ± 22.35		59.38 ± 18.79		56.25 ± 13.56		53.75 ± 12.17	0.495[c]
Change from pre dilution (95% CI)[a]	11.88 (1.23 , 22.52)	0.029	8.75 (-1.89 , 19.39)	0.107	5.00 (-5.64 , 15.64)	0.357	-14.38 (-25.02 , -3.73)	0.008
Difference from heparin-free (95% CI)[b]	26.25 (12.03 , 40.47)	<0.001	23.13 (8.90 , 37.35)	0.001	19.38 (5.15 , 33.60)	0.008	Ref.	

[a] Absolute difference is the mean change from pre dilution with 95%CIs estimated by a linear mixed-effects model adjusted for baseline.

[b] Absolute difference is the mean difference from heparin-free with 95%CIs estimated by a linear mixed-effects model adjusted for baseline.

Information Classification: General

[c] Different time point estimated by One-Way repeated measures ANOVA

Significant if $p<0.05$

Information Classification: General
